# Persistent janus kinase‐signaling in chronic lymphocytic leukemia patients on ibrutinib: Results of a phase I trial

**DOI:** 10.1002/cam4.2042

**Published:** 2019-03-07

**Authors:** David E. Spaner, Lindsay McCaw, Guizhei Wang, Hubert Tsui, Yonghong Shi

**Affiliations:** ^1^ Biology Platform Sunnybrook Research Institute Toronto Canada; ^2^ Department of Medical Biophysics University of Toronto Toronto Canada; ^3^ Sunnybrook Odette Cancer Center Toronto Canada; ^4^ Department of Medicine University of Toronto Toronto Canada; ^5^ Department of Immunology University of Toronto Toronto Canada; ^6^ Division of Hematopathology Sunnybrook Health Sciences Center Toronto Canada; ^7^ Department of Laboratory Medicine and Pathobiology University of Toronto Toronto Canada

**Keywords:** chemokines, chronic lymphocytic leukemia, ibrutinib, janus kinases, ruxolitinib, thrombopoiesis

## Abstract

Methods to deepen clinical responses to ibrutinib are needed to improve outcomes for patients with chronic lymphocytic leukemia (CLL). This study aimed to determine the safety and efficacy of combining a janus kinase (JAK)‐inhibitor with ibrutinib because JAK‐mediated cytokine‐signals support CLL cells and may not be inhibited by ibrutinib. The JAK1/2 inhibitor ruxolitinib was prescribed to 12 CLL patients with abnormal serum beta‐2 microglobulin levels after 6 months or persistent lymphadenopathy or splenomegaly after 12 months on ibrutinib using a 3 + 3 phase 1 trial design (NCT02912754). Ibrutinib was continued at 420 mg daily and ruxolitinib was added at 5, 10, 15, or 20 mg BID for 3 weeks out of five for seven cycles. The break was mandated to avoid anemia and thrombocytopenia observed with ruxolitinib as a single agent in CLL. The combination was well‐tolerated without dose‐limiting toxicities. Cyclic changes in platelets, lymphocytes, and associated chemokines and thrombopoietic factors were observed and partial response criteria were met in 2 of 12 patients. The results suggest that JAK‐signaling helps CLL cells persist in the presence of ibrutinib and ruxolitinib with ibrutinib is well‐tolerated and may be a useful regiment to use in combination therapies for CLL.

## 1 INTRODUCTION

The Bruton's tyrosine kinase (BTK) inhibitor ibrutinib has been a major advance in chronic lymphocytic leukemia (CLL) therapy but it does not cure as a single agent and outcomes for patients who develop progressive disease on ibrutinib are poor. Novel approaches are needed to improve the depth of clinical responses to ibrutinib.^1^, ^2^

The pathogenic importance in CLL of B‐cell receptor (BCR)‐ and toll‐like receptor (TLR)‐signals that employ BTK is emphasized by the remarkable activity of ibrutinib.^1^, ^3^ Growth and survival of CLL cells are also affected by cytokines that signal through ibrutinib‐insensitive janus kinases (JAKs).^4^, ^5^ Plasma levels of many cytokines are reduced by ibrutinib in CLL patients but IL4, IL6, and others are not changed significantly,^6^ could continue to support CLL cells, and lead ultimately to disease progression. Consistent with this idea, IL4 signals through JAK1 and JAK3 reduce the activity of BTK‐inhibitors in vitro, which can be restored with the JAK1/3 inhibitor tofacitinib.^7^ JAK inhibitors might then improve responses to ibrutinib in CLL patients.

Ruxolitinib is a JAK1/2 inhibitor approved for the treatment of intermediate or high‐risk myelofibrosis and polycythemia vera after an inadequate response or intolerance to hydroxyurea.^8^, ^9^ In CLL, it has relatively weak therapeutic activity and can cause significant anemia and thrombocytopenia as a single agent.^10^, ^11^ However, when combined with ibrutinib, it sensitizes CLL cells to cytotoxic drugs in vitro.^5^

These considerations motivated a phase I trial to characterize the toxicity and therapeutic activity of combining ibrutinib with ruxolitinib. Ruxolitinib was administered on a discontinuous schedule to ameliorate potential problems with anemia and thrombocytopenia.^10^ The study population consisted of patients with relapsed/refractory CLL who had not achieved complete remission (CR) after 1 year of ibrutinib. Patients with elevated levels of plasma β2M after 6 months on ibrutinib were also included, since failure to normalize β2M in this time is associated with shorter progression free survival.^13^

## 2 METHODS

### 2.1 Study design and participants

This was a single center phase I study to determine the toxicity of combining ruxolitinib with ibrutinib in CLL patients. Eligible patients were males or females currently treated with ibrutinib at a daily dose of 420 mg due to relapsed/refractory CLL or primary del17p cytogenetic lesions and: (1) failure of plasma β2M levels to decrease below 2.5 μg/L within 6 months of starting ibrutinib or (2) persistent lymphocytosis (>5 × 10^6^ cells/L) and splenomegaly (>11.5 cm)^14^ or lymphadenopathy (marker node >1.5 cm on CT scans) after 1 year on ibrutinib.^1^, ^13^ Exclusion criteria included inadequate bone marrow reserve indicated by neutrophils less than 0.75 × 10^9^/L, platelets less than 75 × 10^9^/L without the assistance of growth factors, thrombopoietic factors, or platelet transfusions, or hemoglobin less than 65 g/L despite transfusions.

The study was approved by the Sunnybrook Research Ethics Board and Health Canada and conducted according to the principles of the Declaration of Helsinki and the Guidelines for Good Clinical Practice. All patients provided written informed consent.

### 2.2 Procedures

Ibrutinib was taken continuously at 420 mg daily. Ruxolitinib was administered over a 35‐day treatment cycle repeated seven times. Each cycle consisted of 3 weeks of ruxolitinib followed by 2 weeks off. The rationale for this discontinuous schedule came from a prior phase II trial in previously untreated CLL patients^10^ where ruxolitinib as a single agent caused severe anemia and thrombocytopenia that tended to develop after 3‐4 weeks and reversed within 2‐3 weeks off treatment. Discontinuous use of ruxolitinib did not seem to greatly impact therapeutic efficacy and was incorporated into the current trial to avoid anticipated hematopoietic problems.

The trial involved 12 patients in a typical 3 + 3 phase I design.^15^ The first cohort of three patients started at 5 mg BID which is the lowest recommended dose for myelofibrosis and polycythemia vera^8^, ^9^ and also had some clinical activity as a single agent in CLL.^10^ Cohorts 2, 3, and 4 were treated at 10, 15, and 20 mg BID, assuming no dose‐limiting toxicities (DLTs) were experienced during the first treatment cycle. Based on the prior phase II trial of single agent ruxolitinib,^10^ it was felt there would be no need to explore doses greater than 20 mg BID and therapeutic effects were anticipated in each group.

Therapeutic activity of the combination was evaluated after seven cycles based on an average time to the best response of 7.4 months associated with other kinase inhibitors including ibrutinib.^1^, ^10^ Ruxolitinib was expected to deepen the response to ibrutinib. If no improvement was seen after seven cycles, it would be concluded ruxolitinib did not fulfill this expectation. Based on Gehan criteria,^16^ a new drug or drug combination must show activity in at least 1 of 13 patients to justify further testing.

The primary endpoint was to determine the maximum tolerated dose (MTD) of ruxolitinib in combination with ibrutinib. The secondary endpoints were safety and tolerability of the combination and overall response rate (ORR), defined as the proportion of subjects with complete or partial responses according to the NCI‐WG guidelines on CLL.^17^

Patients were monitored by history, physical examination, and blood tests on days 1 and 21 of each cycle and for 1 month following completion of treatment. Adverse events were graded according to the Common Toxicity Criteria of the National Cancer Institute, Version 4.0. Hematological toxicities were graded according to IWCLL 2008 criteria.^17^ Response assessments by CT scans were performed after seven cycles of ruxolitinib or earlier if indicated clinically. Bone marrow aspirates and biopsies were taken prior to study entry, at day 21 of cycle 3 (C3D21) prior to the rest period for that cycle and at the end of treatment (EOT). Responses were assessed by IWCLL guidelines.^14^, ^17^

Exploratory endpoints included measurements of 42 plasma cytokines, chemokines, and growth factors at the beginning of a treatment cycle when ruxolitinib had not been taken for at least 14 days and again at day 21 of ruxolitinib, prior to a rest period. In addition, 64 proteins were measured in bone marrow plasma obtained prior to starting ruxolitinib and again at C3D21. Due to the nature of the study, statistical analysis was mainly descriptive.

The study was registered with ClinicalTrials.gov, number NCT02912754.

### 2.3 Cell and plasma preparation

CLL cells were isolated from blood and bone marrow samples by negative selection and density gradient centrifugation as before.^4^, ^10^ Aliquots were cryopreserved. Plasma was aliquoted and stored at −80°C.

### 2.4 Serum β2M and complete blood counts

Serum β2M was measured by the clinical service laboratory. Blood hemoglobin (Hb), platelets, and lymphocyte counts were determined in the clinical hematology laboratory at Sunnybrook. Results were obtained from the patient's electronic medical record.

### 2.5 Flow cytometry

Blood samples were enumerated by a hematology analyzer Beckman Coulter (BC) (Fullerton, CA) in the Sunnybrook clinical hematology laboratory. White blood cells (WBCs) were adjusted to 1 × 10^7^ cells/mL and 100 μL (1 × 10^6^ cells) was stained with antibody combinations. Red blood cells were lysed with Versalyse^™^ (BC). Samples were run on a 10 color Navios^™^ (BC) flow cytometer and analyzed with Kaluza^™^ software (BC). Antibody combinations consisted of a screening panel of 14 antibodies (CD3, CD4, CD5, CD8, CD10, CD14, CD19, CD20, CD33, CD34, CD45, CD56, kappa Ig light chain, and lambda Ig light chain) or a lymphoproliferative panel of 18 antibodies (CD2, CD3, CD4, CD5, CD7, CD8, CD10, CD11c, CD19, CD20, CD23, CD34, CD38, CD45, CD56, CD57, kappa, and lambda). Unless otherwise specified, a minimum of 5 × 10^4^ leukocytes were acquired per patient sample.

### 2.6 Plasma protein measurements

Analysis of 42 different analytes in a “42‐plex Discovery platform” was performed as before by Eve Technologies (Calgary, AB, Canada) using Multiplexing LASER Bead Technology.^10^ Proteins measured included EGF, Eotaxin, FGF2 (basic), Flt‐3 ligand, G‐CSF, GM‐CSF, GRO (CXCL1), IFNα2, IFNγ, IL‐1‐10, IL‐12, IL‐13, IL‐15, IL‐17, IL‐18 IP‐10, MCP‐1, MCP‐3, MDC, MIP‐1α, MIP‐1β, PDGF‐AA, PDGF‐BB, RANTES, CD40L, TNFα, TNFβ, and VEGF. An additional 23 analytes including thrombopoietin (TPO) were measured with a “65‐plex Discovery platform” in bone marrow plasma. Concentrations were determined from standard curves. The assays were linear between 30 and 1000 pg/mL of cytokine.

### 2.7 Role of the funding source

This was an investigator‐initiated and sponsored trial supported by Novartis. The investigator was responsible for the study design and analysis plan. The company was apprised regularly of adverse events as well as the progress of the trial but the corresponding author is fully responsible for the accuracy of the data and conclusions reported in this manuscript.

## 3 RESULTS

### 3.1 Patients

Between June 6 and October 30, 2017, 12 CLL patients with measurable disease after 1 year or elevated β2M levels after 6 months on ibrutinib were enrolled in the study. Patient characteristics are described in Table 1.

**Table 1:** Patient data

Patient No.	Sex	Age	Time^a^ (years)	C1D1 lymphocytes (×10^9^/L)	CD38 (%)	C1D1 β2m^b^	FISH^c^	Tx^d^	Prior ibrutinib use (months)
Group 1									
JAK2001	M	73	13	3.1	24	2.2	13q,17p	6	15
JAK2002	F	63	5	0.8	93	2	11q	3	29
JAK2003	M	61	17	1.7	20	3.2	Normal	9	34
Group 2									
JAK2005	F	71	10	8	100	1.8	13q	3	15
JAK2007	F	81	10	1.4	1	3.7	13q,17p	2	28
JAK2008	M	62	12	2.5	1	2.6	T12^e^	4	9
Group 3									
JAK2009	M	70	7	0.7	1	2.2	Normal	3	15
JAK2010	M	70	12	5.3	1	2.1	13q	4	27
JAK2011	M	80	12	0.9	12	2.8	11q,T12	3	14
Group 4									
JAK2012	F	79	10	1.4	18	3	T12,17p	2	36
JAK2014	M	64	22	1.3	23	1.8	13q,17p	5	22
JAK2015	M	78	9	0.8	27	3.4	13q,11q	4	22

a Time since initial diagnosis.

b Normal range: 0.6‐2.3 μg/mL.

c Fluorescence in situ hybridization.

d Number of treatments including alkylator and fludarabine regimens, splenectomy, and ibrutinib.

e T12 = trisomy 12.

### 3.2 Toxicity

The addition of ruxolitinib to ibrutinib was generally well‐tolerated by all four cohorts. Four patients did not complete the seven mandated cycles of ruxolitinib. JAK2003 in group 1 (5 mg ruxolitinib) had evidence of CLL progression after five cycles and was removed from the trial for salvage therapy. This patient had progressive lymphadenopathy on ibrutinib prior to entering the trial. JAK2011 in group 3 (15 mg ruxolitinib) was removed from the study during cycle 5 due to a squamous cell skin cancer that required radiation. It was not considered related to ruxolitinib because he had a history of such tumors. JAK2012 in group 4 (20 mg ruxolitinib) asked to be withdrawn from the trial after cycle 3 due to the inconvenience of the frequent clinic visits. JAK2015 in group 4 did not receive the final cycle of ruxolitinib to allow him to recover from a mild viral illness contracted during a holiday while on the 2‐week rest period following cycle 6. It was felt that negligible additional information would be gained by prescribing cycle 7. JAK2014 in group 4 had a retroperitoneal mass that was presumed to be lymphomatous at study entry. The mass was larger upon completing the trial but a subsequent biopsy showed it to be a spindle cell tumor that is simply being observed at this time.

**Figure 1:**
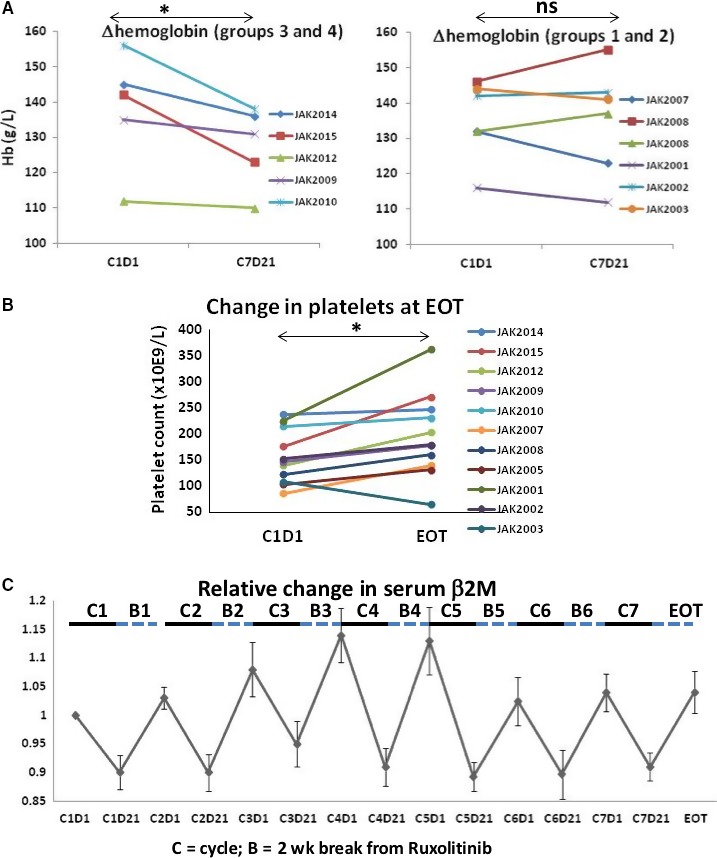
Effect of ruxolitinib on hemoglobin (Hb), platelets, and β2M. (A) Hb was measured before starting ruxolitinib while on ibrutinib (C1D1) and after completing the seventh cycle of ruxolitinib (C7D21). The lines indicate results for individual patients and data for groups 1 and 2 (5 and 10 mg ruxolitinib) and groups 3 and 4 (15 and 20 mg ruxolitinib) are shown in different graphs. (B) Platelet counts were measured at C1D1 and at end of treatment (EOT). (C) β2M was measured at each time‐point and normalized to the initial value at C1D1. The average and standard error of these ratios for all patients are plotted as a function of time and show that β2M levels decreased during treatment with ruxolitinib (indicated by the solid bars labeled “C”) but recovered during the 2 week break from ruxolitinib in each cycle (indicated by the dashed bars labeled “B”). *P < 0.05; ns, not significant

No DLTs or adverse events greater than grade 2 were observed in the trial. Symptomatic anemia, a major issue with ruxolitinib as a single agent in CLL,^10^ was not a problem (Figure 1A). However, at higher doses of ruxolitinib (groups 3 and 4), hemoglobin levels decreased as much as 20 g/L in some patients after seven treatment cycles (ie, JAK2010 and JAK2015) (Figure 1A). Thrombocytopenia was also seen with single agent ruxolitinib^10^ but not in combination with ibrutinib. In fact, platelets increased in most patients after seven cycles of ruxolitinib except for JAK2002 with disease progression (Figure 1B).

Consideration of the toxicity data, especially the decline in Hb (Figure 1A), identified a ruxolitinib dose of 15 mg BID as suitable for subsequent clinical trials with ibrutinib.

### 3.3 Clinical responses

The trial design and nature of disease progression on ibrutinib meant tumor burdens were low in most patients upon initiating ruxolitinib. Regardless, ruxolitinib produced partial responses (PRs) in two patients (JAK2001 and JAK2007), based on marker lymph node diameters and bone marrow CLL cell percentages that decreased more than 50% (Table 2). Two patients (JAK2002 and JAK2003) exhibited progressive disease (PD) on ruxolitinib. Both had demonstrated evidence of disease progression on ibrutinib alone and were treated with the lowest dose of ruxolitinib (5 mg BID) in group 1. The remaining patients were classed as having stable disease (SD) since residual lymphadenopathy, splenomegaly, or bone marrow involvement did not change by more than 50%.^18^ However, marker lymph nodes and/or splenomegaly decreased at EOT in JAK2005, JAK2008, JAK2009, JAK2011, JAK2014, and JAK2015 (Table 2), although not enough to meet the requirement for PR.

**Table 2:** Response data

Patient No.	Cycles	Bone marrow CLL cells (%)			Marker LN (cm)		Spleen size (cm)		Response^e^
		C1D1	C3D21	EOT	C1D1	EOT	C1D1	EOT	
Group 1									
JAK2001	7	40	25	18	1.5	0.7	NA	NA	PR
JAK2002	7	6	25	46	2.2	2.7	11.7	11.1	PD
JAK2003	5^a^	4	9	NA	3.6	9.7	12.5	13.3	PD
Group 2									
JAK2005	7	58	68	54	0.8	0.7	12.2	11.9	SD
JAK2007	7	9	8	1	2.8	2.3	14.8	12.6	PR
JAK2008	7	15	13	23	1.8	1.6	11.5	11.6	SD
Group 3									
JAK2009	7	0	0	0	0.9	0.8	13.1	12.4	SD
JAK2010	7	4	2	3	0.9	0.9	NA	NA	SD
JAK2011	5^b^	7	24	NA	1.5	0.7	10.6	10.6	SD
Group 4									
JAK2012	3^c^	2	4	NA	0.8	0.8	9.4	9.4	SD
JAK2014	7	5	7	2.9	0.9	0.9	13.3	12.7	SD
JAK2015	6^d^	0	0	NA	1.8	1.8	12.9	12.3	SD

NA, not available.

a Discontinued due to disease progression.

b Stopped 1 week into cycle 5 due to skin cancer and radiation therapy.

c Stopped after three cycles at patient's request.

d Cycle 7 not given at discretion of patient and investigator.

e PR = partial response, PD = progressive disease, SD = stable disease.

High blood levels of β2M are associated with progressive CLL.^13^, ^18^ The clearance of residual disease by ruxolitinib was expected to be associated with normalization of β2M levels. Regardless of the initial levels (Table 1), β2M decreased in all patients during each cycle on ruxolitinib (Figure 1C). However, β2M levels increased during the 2‐week break from ruxolitinib that was incorporated into each cycle and there was no significant change at EOT (Figure 1C).

### 3.4 Changes in blood lymphocytes

**Figure 2:**
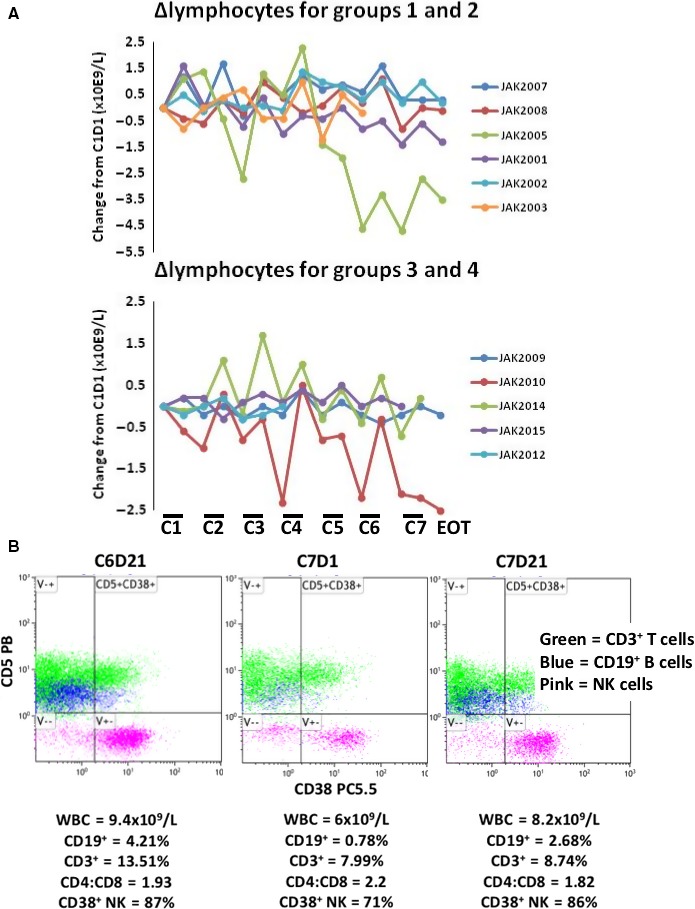
Effect of ruxolitinib on blood lymphocytes. (A) Lymphocyte numbers at each time‐point were taken from the medical record. Differences between lymphocyte counts at each time‐point and the initial count at C1D1 were plotted as a function of time. Each line represents results for an individual patient. Groups 1 and 2 are distinguished from groups 3 and 4. The bars labeled C1, C2, C3, etc. represent the 3‐week period on ruxolitinib during each treatment cycle. Results for JAK2011 were considered uninterpretable as a result of confounding clinical events not related to the trial and were not included in the analysis. (B) Blood from JAK2014 was collected at C6D21, C7D1, and C7D21 and analyzed by 10‐color flow cytometry. Percentages of selected lymphocyte populations at each time‐point are shown below the respective dot‐plots and suggest CD5^+^ CD19^+^ CLL cells and CD38^+^ NK cells cycle in and out of the blood in the presence and absence of ruxolitinib

Similar to β2M (Figure 1C), lymphocytes also cycled (Figure 2A). Blood lymphocytes generally increased on ruxolitinib but decreased during each scheduled 2‐week break from ruxolitinib (Figure 2A). Initial lymphocyte numbers were in or below the normal range and did not change significantly at EOT in most patients. In the two patients with persistent lymphocytosis on ibrutinib (JAK2010 and JAK2005 (Table 1)), blood lymphocytes decreased at EOT.

Detailed analysis of changes in lymphocyte subsets was limited as the cyclical changes caused by ruxolitinib were not appreciated until late in the course of the trial. However, 10‐color flow cytometry was performed on blood from JAK2014 on several occasions. CD5^+^CD19^+^ CLL cells increased following treatment with ruxolitinib (C6D21 and C7D21) and then decreased during the 2‐week period off ruxolitinib (C7D1) (Figure 2B).

Ruxolitinib also appeared to affect other lymphocyte sub‐types. For example, circulating CD38^+^ natural killer (NK) cells increased on ruxolitinib and decreased when ruxolitinib was held (Figure 2B). Changes in neutrophil and monocyte numbers in response to ruxolitinib were not as uniform as changes in blood lymphocytes (not shown).

### 3.5 Effect on platelets

**Figure 3:**
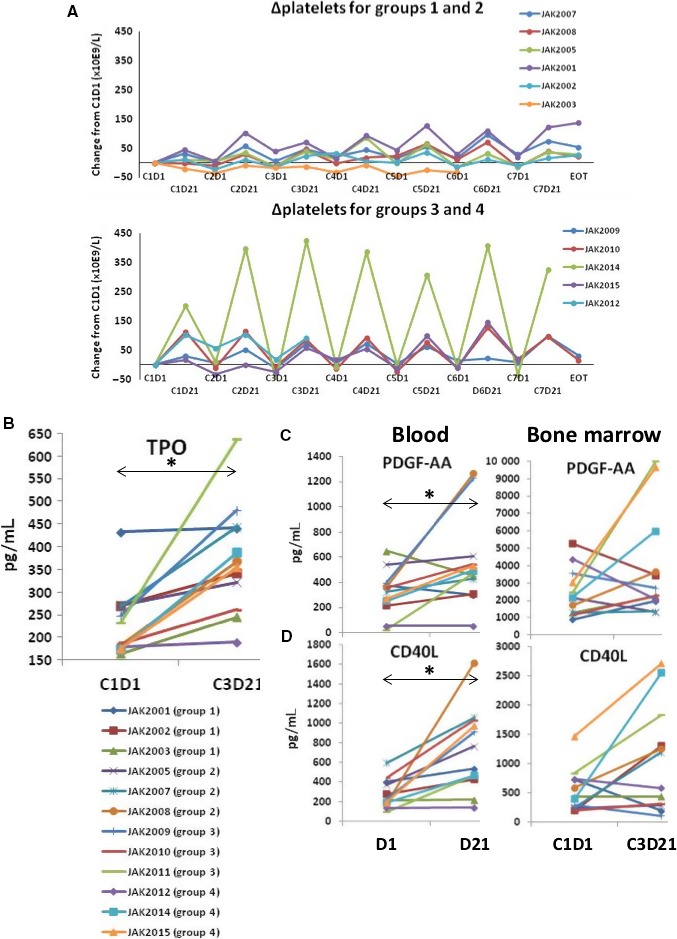
Effect of ruxolitinib on platelets and thrombopoietic cytokines. (A) Platelet counts were taken from the medical record. Differences between the number at each time‐point and initial platelet count at C1D1 were plotted as a function of time. Each line represents the calculations for a single patient. Results for groups 1 and 2 are shown separately from groups 3 and 4. (B‐D) TPO (B), PDGF‐AA (C, right panel), and CD40L (D, right panel) were measured in plasma from bone marrow aspirates obtained at C1D1 and CD3D21. PDGF‐AA (C, left panel) and CD40L (D, left panel) were also measured in plasma from blood at day 1 and day 21 of a treatment cycle. Each line represents results for an individual patient. *P < 0.05

Ruxolitinib in the context of ibrutinib produced an unexpected but remarkable effect on platelets (Figure 3). In all patients, platelet counts increased on ruxolitinib, sometimes by as much as 400 × 10^9^/L in the case of JAK2014 (Figure 3A), and then reduced to near baseline in the rest period off ruxolitinib in each cycle. This effect was seen with all doses of ruxolitinib but appeared somewhat more marked in groups 3 and 4 (15 and 20 mg ruxolitinib) (Figure 3A).

These cyclic changes in platelet numbers could reflect increased thrombopoiesis, decreased clearance by macrophages, or both. In support of an effect on thrombopoiesis, thrombopoietin (TPO) levels in bone marrow plasma increased in all patients at C3D21 compared to baseline levels (Figure 3B). Platelet‐derived growth factor (PDGF) is associated with megakarycytopoiesis^19^ and PDGF‐BB levels increased in blood and bone marrow plasma at C3D21 compared with baseline in most patients (Figure 3C). Plasma CD40L is also associated with platelet regeneration^20^ and increased in blood and bone marrow plasma at C3D21 (Figure 3D).

### 3.6 Effect on cytokines and chemokines

**Figure 4:**
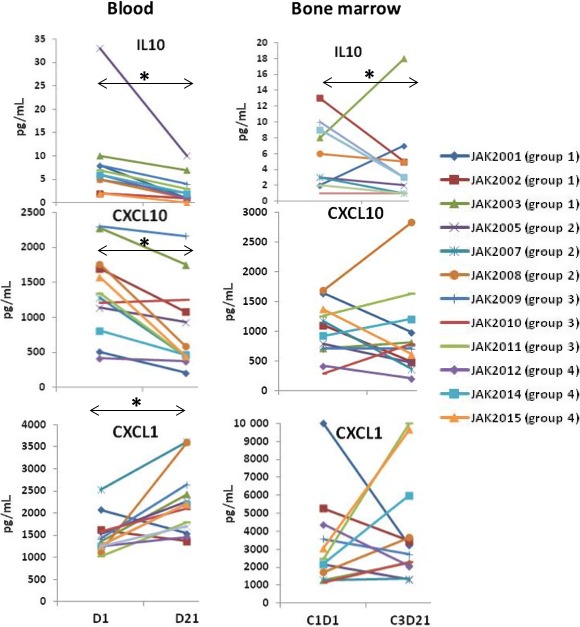
Effect of ruxolitinib on IL10, CXCL10, and CXCL1. IL10, CXCL10, and CXCL1 were measured in plasma from blood at day 1 and day 21 of a treatment cycle (left panels) and also in plasma from marrow aspirates obtained at C1D1 and CD3D21 (right panels). Each line represents results for an individual patient. *P < 0.05

Ruxolitinib decreases IL10 production by activated macrophages,^21^ which may help indicate on‐target activity of this drug in CLL patients. IL10 levels are also reduced by ibrutinib^22^ and were relatively low at baseline. However, ruxolitinib further decreased IL10 in blood and bone marrow plasma in almost all patients (Figure 4A).

Compartmental shifts in lymphocytes that were characteristic of treatment with the combination of ruxolitinib and ibrutinib (Figure 2) are often due to changes in chemokine gradients. CXLCL10 is a chemoattractant for T, NK, and B cells^23^ and CXCL1 affects some T‐cell subsets although it attracts mainly neutrophils.^24^ Ruxolitinib generally decreased CXCL10 and increased CXCL1 in the blood with more variable effects in bone marrow (Figure 4B). These findings suggested chemokine gradients that could affect lymphocyte trafficking were altered by ruxolitinib.

## 4 DISCUSSION

The results of this trial suggest the combination of ruxolitinib and ibrutinib is well‐tolerated and establish 15 mg PO BID of ruxolitinib as the dose for future trials. The combination also appears to have therapeutic activity since 2 of 12 patients exhibited PRs after seven cycles of treatment, exceeding the threshold of 1 of 13 established by Gehan to warrant further investigation.^16^ However, the therapeutic effects were relatively modest and the regimen designed for this trial is unlikely to serve as a “stand‐alone” treatment for CLL.

The median time to the best response with ibrutinib in CLL patients is 7.4 months but the quality evolves with time and median time to CR is 21.1 months.^1^ It is possible that apparent responses with addition of ruxolitinib were simply due to continued use of ibrutinib. However, JAK2007 had been on ibrutinib for 28 months but still exhibited a partial response to ruxolitinib (Tables 1 and 2). Similarly, JAK2014 and JAK2015 had been treated with ibrutinib for 22 months (Table 1) but their spleen sizes decreased by 0.6 cm following treatment with ruxolitinib (Table 2). Ibrutinib and ruxolitinib are also both metabolized by the Cyp3A4 system and Cyp3A4 inhibitors increase the concentration of ibrutinib without increasing the half‐life.^12^ It is then also possible that adding ruxolitinib simply increased plasma concentrations of ibrutinib to account for improved response quality. A randomized trial comparing outcomes with ibrutinib and ruxolitinib to ibrutinib alone is needed to properly assess the additional therapeutic benefit of ruxolitinib.

Why was the combination of ruxolitinib and ibrutinib not more effective and why did no patients enter complete remissions (CRs)? While only two patients (JAK2001 and JAK2008) met criteria for PRs at EOT, six more (JAK2007, JAK2008, JAK2009, JAK2011, JAK2014, and JAK2015) showed diminution of residual lymphadenopathy and splenomegaly (Table 2). It is possible more profound responses including CRs may have occurred simply by continuing ruxolitinib and ibrutinib for longer than 7 cycles.

Alternatively, therapeutic responses may have been weakened by the length of time off ruxolitinib in each treatment cycle. β2M in blood, a marker of tumor burden,^13^ decreased during each treatment cycle but recovered in the 2‐week break from ruxolitinib (Figure 1C). This observation suggests β2M levels in CLL patients reflect pathological JAK‐mediated cytokine activity, although the cellular origin of β2M is unclear and may not necessarily be from CLL cells. The discontinuous schedule was instituted to avoid anticipated problems with anemia and thrombocytopenia but may have inadvertently allowed partial reversal of the therapeutic gains achieved by adding ruxolitinib to ibrutinib. Since anemia and thrombocytopenia were not problematic (Figure 1A and B), the efficacy may be improved by shortening the period off ruxolitinib.

Due to the design of the trial, patients were included who had already been on ibrutinib for many months (Table 1). These patients may have harbored residual leukemia cells that had lost sensitivity to ibrutinib. Such cells would effectively only experience the inhibitory activity of ruxolitinib alone, which was shown previously to be rendered ineffective by compensatory BTK/NFκB‐mediated signaling pathways.^5^, ^10^ JAK2002 and JAK2003 who experienced disease progression on ruxolitinib (Table 2) had developed increased adenopathy on ibrutinib and would likely not have derived any benefit from simultaneous blockade of BTK/NFκB and JAK/STAT signaling pathways. This problem might be dealt with by adding ruxolitinib at the same time as ibrutinib to avoid prior development of ibrutinib‐resistance.

The patients who exhibited PRs in response to adding ruxolitinib (JAK2007 and JAK 2001) harbored 13q and 17p deletions in their leukemia cells (Tables 1 and 2). JAK2014 had a similar cytogenetic profile and also exhibited a decrease in bone marrow CLL cells and spleen size at the end of treatment with ibrutinib and ruxolitinib (Tables 1 and 2). It has been recently suggested that p53‐defective CLL cells are less dependent on BCR‐signaling^34^ and we have shown that CLL cells with 17p deletions have exaggerated responses to cytokines like type 1 interferons.^35^ Perhaps the presence of a 17p deletion or p53 mutation is a biomarker for CLL cells that are more dependent on JAK‐signaling and consequently more responsive to the combination of ibrutinib and ruxolitinib.

The reason for anemia from ineffective erythropoiesis was a major problem with ruxolitinib as a single agent in CLL patients^10^ but such a minor problem in this trial (Figure 1A) is unclear. Major differences between the two trials include the burden of leukemia cells, which was much higher in the former trial, and the presence of ibrutinib to block BTK signals and prevent activation of CLL cells in this trial. We have shown ruxolitinib reverses inhibitory effects of cytokines on TLR‐signaling in CLL cells.^4^ Consequently, if ruxolitinib is given to patients with a high tumor burden, it may cause enhanced TLR‐signaling in these cells with the production of cytokines such as TNFα that could inhibit erythropoiesis in the bone marrow.^27^ We speculate this mechanism was not very active in this trial because the number of CLL cells was low and TLR‐signaling was prevented by the presence of ibrutinib.

An unexpected but striking finding was that ruxolitinib increased platelet counts with ibrutinib, in marked contrast to its well‐known effect of causing thrombocytopenia as a single agent.^8^, ^10^ Ibrutinib affects platelets directly,^1^ which may cause them to be cleared by macrophages more rapidly than normal. It is possible that ruxolitinib with ibrutinib suppresses macrophages and increases platelet numbers in the same way that glucocorticoids increase platelet counts in idiopathic thrombocytopenia (ITP).^28^ The effect may also reflect enhanced thrombopoiesis as bone marrow TPO levels increased in all patients and PDGF levels increased in most (Figure 3C and D). Increased TPO levels with JAK inhibition have been observed in genetically modified mice and explained by failure to internalize and degrade TPO.^29^ The results in mice suggested thrombocytopenia associated with JAK inhibitors reflects JAK2 inhibition in stem cells rather than in megakaryocytes.^29^ Accordingly, it is possible ibrutinib somehow protects stem cells from ruxolitinib in CLL patients. TPO may be able to signal in the presence of ruxolitinib through JAK‐independent pathways in the same way that erythropoietin increased erythropoiesis in CLL patients on ruxolitinib as a single agent.^10^ Alternatively, blood levels of IL10 decreased in all patients on ruxolitinib (Figure 4A). Given that IL10 can suppress thrombopoiesis,^30^ the increase in platelets may reflect abrogation of its negative effects. Because the positive effect of ruxolitinib and ibrutinib on platelets was seen in all patients, including those without detectable bone marrow leukemia cells (ie, JAK2009 and JAK2015 (Table 2)), it may not be unique to CLL and could potentially be exploited in other conditions with problematic thrombocytopenia such as aplastic anemia, ITP, and myelodysplastic syndrome (MDS).

Ruxolitinib and ibrutinib appear to be a well‐tolerated combination that may be useful for CLL patients if maintained for longer than 7 months and with more continuous administration of the JAK inhibitor. However, the utility of this regimen may lie in its ability to enhance therapeutic responses to cytotoxic drugs.^5^, ^31^ Neither ibrutinib, ruxolitinib, nor the combination is cytotoxic to CLL cells in vitro^5^ and it is not well‐understood how CLL cells are eliminated by ibrutinib in vivo.^25^ Ibrutinib may sensitize CLL cells to cytotoxic stresses in the body by flushing them out of protective microenvironments in part by blocking CXCR4‐signaling.^25^ Since CXCR4 also signals through JAKs,^26^ ruxolitinib may flush additional CLL cells from protective microenvironments. Indeed CD5^+^CD19^+^ CLL cells increased in the blood in the presence of ruxolitinib (Figure 2B) and appeared to exit when ruxolitinib was held, associated with changes in chemokines that mediate lymphocyte trafficking (Figure 4). Perhaps, the addition of a cytotoxic drug would enhance clearance of CLL cells in the presence of ibrutinib and ruxolitinib.

Current trials suggest the most potent therapeutic regimens for CLL will likely involve combinations of ibrutinib and the Bcl‐2 antagonist venetoclax.^32^ Venetoclax is limited by the presence of microenvironmental signals that up‐regulate Bcl‐2 family members it does not target. Ibrutinib inhibits some these signals but performs poorly compared to more nonspecific agents in screens of kinase inhibitors to improve the cytotoxic activity of venetoclax in microenvironmental conditions.^33^ These observations suggest ibrutinib‐insensitive signals will continue to allow a sub‐population of CLL cells to survive in CLL microenvironments that may eventually mediate disease progression despite treatment with venetoclax and ibrutinib. The results of this phase I trial suggest ruxolitinib may be able to flush these otherwise resistant cells out of their protective microenvironments and improve even further the therapeutic activity of venetoclax and ibrutinib regimens.

Ibrutinib and ruxolitinib have been shown to sensitize CLL cells to cytotoxic glucocorticoids such as dexamethasone in vitro.^5^, ^31^ To test the idea that combining ruxolitinib and ibrutinib sensitizes CLL cells to cytotoxic agents more effectively than ibrutinib alone, we have opened a randomized phase II trial comparing dexamethasone and ibrutinib with dexamethasone, ibrutinib, and ruxolitinib in CLL patients with relapsed disease (NCT02912754). In this trial, the break from ruxolitinib has been shortened to 1 week in each cycle to lessen the possibility of regrowth of leukemia cells (Figure 1C).

## CONFLICT OF INTEREST

D. E. Spaner reports grants from Novartis to support the submitted work and personal fees from Janssen outside the submitted work. The other authors declare no conflicts of interest with respect to this work.

## DATA SHARING

Data for this study are the property of the sponsor (DS) and the Sunnybrook Research Institute. Data and related documents to the study can be requested from the corresponding author from the date of publication. The access of data and documents will require the agreement of the sponsor.

## References

[1] Byrd JC , Furman RR , Coutre SE , et al. Three‐year follow‐up of treatment‐naive and previously treated patients with CLL and SLL receiving single‐agent ibrutinib. Blood. 2015;125:2497‐2506. 25700432 10.1182/blood-2014-10-606038 PMC4400288

[2] Pinilla‐Ibarz J , Chavez JC . Life after ibrutinib? A new unmet need in CLL. Blood. 2015;125:2013‐2014. 25814485 10.1182/blood-2015-02-622472

[3] Mertens D , Stilgenbauer S . Ibrutinib‐resistant CLL: unwanted and unwonted!. Blood. 2017;129:1407‐1409. 28302688 10.1182/blood-2017-01-761536

[4] Li Y , Shi Y , McCaw L , et al. Microenvironmental interleukin‐6 suppresses toll‐like receptor signaling in human leukemia cells through miR‐17/19A. Blood. 2015;126:766‐778. 26041742 10.1182/blood-2014-12-618678

[5] Oppermann S , Lam AJ , Tung S , et al. Janus and PI3‐kinases mediate glucocorticoid resistance in activated chronic leukemia cells. Oncotarget. 2016;7:72608‐72621. 27579615 10.18632/oncotarget.11618 PMC5341931

[6] Niemann CU , Herman SE , Maric I , et al. Disruption of in vivo CLL tumor‐microenvironment interactions by ibrutinib ‐ findings from an investigator initiated phase 2 study. Clin Cancer Res. 2015;22:1572‐1582. 26660519 10.1158/1078-0432.CCR-15-1965 PMC4818677

[7] Aguilar‐Hernandez MM , Blunt MD , Dobson R , et al. IL‐4 enhances expression and function of surface IgM in CLL cells. Blood. 2016;127:3015‐3025. 27002119 10.1182/blood-2015-11-682906

[8] Verstovsek S , Mesa RA , Gotlib J , et al. A double‐blind, placebo‐controlled trial of ruxolitinib for myelofibrosis. N Engl J Med. 2012;366:799‐807. 22375971 10.1056/NEJMoa1110557 PMC4822164

[9] Passamonti F , Griesshammer M , Palandri F , et al. Ruxolitinib for the treatment of inadequately controlled polycythaemia vera without splenomegaly (RESPONSE‐2): a randomised, open‐label, phase 3b study. Lancet Oncol. 2017;18:88‐99. 27916398 10.1016/S1470-2045(16)30558-7

[10] Spaner DE , Wang G , McCaw L , et al. Activity of the janus kinase inhibitor Ruxolitinib in CLL: results of a phase II trial. Haematologica. 2016;101:e192‐e195. 26819050 10.3324/haematol.2015.135418 PMC5004376

[11] Jain P , Keating M , Renner S , et al. Ruxolitinib for symptom control in patients with CLL: a single‐group, phase 2 trial. Lancet Haematol. 2017;4:e67‐e74. 28089238 10.1016/S2352-3026(16)30194-6 PMC5356368

[12] de Jong J , Skee D , Murphy J , et al. Effect of CYP3A perpetrators on ibrutinib exposure in healthy participants. Pharmacol Res Perspect. 2015;3:e00156. 26171235 10.1002/prp2.156 PMC4492731

[13] Thompson PA , O'Brien SM , Xiao L , et al. β2‐microglobulin normalization within 6 months of ibrutinib‐based treatment is associated with superior progression‐free survival in patients with chronic lymphocytic leukemia. Cancer. 2016;122:565‐573. 26588193 10.1002/cncr.29794 PMC4813299

[14] Linguraru MG , Sandberg JK , Jones EC , Summers RM . Assessing splenomegaly: automated volumetric analysis of the spleen. Acad Radiol. 2013;20:675‐684. 23535191 10.1016/j.acra.2013.01.011 PMC3945039

[15] Le Tourneau C , Lee JJ , Siu LL . Dose escalation methods in phase I cancer clinical trials. JNCI. 2009;101:708‐720. 19436029 10.1093/jnci/djp079 PMC2684552

[16] Gehan EA . The determination of the number of patients required in a preliminary and a follow‐up trial of a new chemotherapeutic agent. J Chronic Dis. 1961;13:346‐353. 13704181 10.1016/0021-9681(61)90060-1

[17] Hallek M , Cheson BD , Catovsky D , et al. International workshop on chronic lymphocytic leukemia. Guidelines for the diagnosis and treatment of chronic lymphocytic leukemia: a report from the International Workshop on Chronic Lymphocytic Leukemia updating the National Cancer Institute‐Working Group 1996 guidelines. Blood. 2008;111:5446‐5456. 18216293 10.1182/blood-2007-06-093906 PMC2972576

[18] International CLL‐IPI Working Group . An international prognostic index for patients with chronic lymphocytic leukaemia (CLL‐IPI): a meta‐analysis of individual patient data. Lancet Oncol. 2016;17:779‐790. 27185642 10.1016/S1470-2045(16)30029-8

[19] Yang M , Chesterman CN , Chong BH . Recombinant PDGF enhances megakaryocytopoiesis in vitro. Br J Haematol. 1995;91:285‐289. 8547063 10.1111/j.1365-2141.1995.tb05291.x

[20] Viallard JF , Solanilla A , Gauthier B , et al. Increased soluble and platelet‐associated CD40 ligand in essential thrombocythemia and reactive thrombocytosis. Blood. 2002;99:2612‐2624. 11895803 10.1182/blood.v99.7.2612

[21] Pattison MJ , Mackenzie KF , Arthur JS . Inhibition of JAKs in macrophages increases lipopolysaccharide‐induced cytokine production by blocking IL‐10‐mediated feedback. J Immunol. 2012;189:2784‐2792. 22904308 10.4049/jimmunol.1200310 PMC3443740

[22] Long M , Beckwith K , Do P , et al. Ibrutinib treatment improves T cell number and function in CLL patients. J Clin Invest. 2017;127:3052‐3064. 28714866 10.1172/JCI89756 PMC5531425

[23] Antonelli A , Ferrari SM , Giuggioli D , Ferrannini E , Ferri C , Fallahi P . Chemokine (C‐X‐C motif) ligand (CXCL)10 in autoimmune diseases. Autoimmun Rev. 2014;13:272‐280. 24189283 10.1016/j.autrev.2013.10.010

[24] Lv M , Xu Y , Tang R , et al. miR141‐CXCL1‐CXCR2 signaling‐induced Treg recruitment regulates metastases and survival of non‐small cell lung cancer. Mol Cancer Ther. 2014;13:3152‐3162. 25349304 10.1158/1535-7163.MCT-14-0448

[25] Burger JA , Montserrat E . Coming full circle: 70 years of CLL cell redistribution, from glucocorticoids to inhibitors of B‐cell receptor signaling. Blood. 2013;121:1501‐1509. 23264597 10.1182/blood-2012-08-452607 PMC4968370

[26] Ahr B , Denizot M , Robert‐Hebmann V , Brelot A , Biard‐Piechaczyk M . Identification of the cytoplasmic domains of CXCR4 involved in Jak2 and STAT3 phosphorylation. J Biol Chem. 2005;280:6692‐6700. 15615703 10.1074/jbc.M408481200

[27] Johnson CS , Cook CA , Furmanski P . In vivo suppression of erythropoiesis by tumor necrosis factor‐alpha (TNF‐alpha): reversal with exogenous erythropoietin (EPO). Exp Hematol. 1990;18:109‐113. 2303102

[28] Mizutani H , Furubayashi T , Imai Y , et al. Mechanisms of corticosteroid action in immune thrombocytopenic purpura (ITP): experimental studies using ITP‐prone mice, (NZW x BXSB) F1. Blood. 1992;79:942‐947. 1737103

[29] Meyer SC , Keller MD , Woods BA , et al. Genetic studies reveal an unexpected negative regulatory role for Jak2 in thrombopoiesis. Blood. 2014;124:2280‐2294. 25115888 10.1182/blood-2014-03-560441 PMC4183987

[30] Sosman JA , Verma A , Moss S , et al. Interleukin 10‐induced thrombocytopenia in normal healthy adult volunteers: evidence for decreased platelet production. Br J Haematol. 2000;111:104‐111. 11091188 10.1046/j.1365-2141.2000.02314.x

[31] Shi Y , Wang G , Muhowski EM , et al. Ibrutinib reprograms the glucocorticoid receptor in CLL cells. Leukemia. 2019 [Epub ahead of print]. 10.1038/s41375-019-0381-4. 30696950

[32] Rogers KA , Huang Y , Ruppert AS , et al. Phase 1b study of obinutuzumab, ibrutinib, and venetoclax in relapsed and refractory chronic lymphocytic leukemia. Blood. 2018;132:1568‐1572. 30111609 10.1182/blood-2018-05-853564 PMC6182267

[33] Oppermann S , Ylanko J , Shi Y , et al. High‐content screening identifies kinase inhibitors that overcome venetoclax resistance in activated CLL cells. Blood. 2016;128:934‐947. 27297795 10.1182/blood-2015-12-687814 PMC5000578

[34] Guarini A , Peragine N , Messina M , et al. Unravelling the suboptimal response of TP53‐mutated chronic lymphocytic leukaemia to ibrutinib. Br J Haematol. 2019;184:392‐396. 30338509 10.1111/bjh.15613

[35] Tomic J , Lichty B , Spaner DE . Aberrant interferon‐signaling is associated with aggressive chronic lymphocytic leukemia. Blood. 2011;117:2668‐2680. 21205928 10.1182/blood-2010-05-285999

